# Cross-Species Transmission of a Novel Adenovirus Associated with a Fulminant Pneumonia Outbreak in a New World Monkey Colony

**DOI:** 10.1371/journal.ppat.1002155

**Published:** 2011-07-14

**Authors:** Eunice C. Chen, Shigeo Yagi, Kristi R. Kelly, Sally P. Mendoza, Nicole Maninger, Ann Rosenthal, Abigail Spinner, Karen L. Bales, David P. Schnurr, Nicholas W. Lerche, Charles Y. Chiu

**Affiliations:** 1 Department of Laboratory Medicine, University of California San Francisco, San Francisco, California, United States of America; 2 UCSF-Abbott Viral Diagnostics and Discovery Center, University of California San Francisco, San Francisco, California, United States of America; 3 Viral and Rickettsial Disease Laboratory, California Department of Public Health, Richmond, California, United States of America; 4 California National Primate Research Center, University of California Davis, Davis, California, United States of America; 5 Department of Psychology, University of California Davis, Davis, California, United States of America; 6 Department of Medicine, Division of Infectious Diseases, University of California San Francisco, San Francisco, California, United States of America; The Scripps Research Institute, United States of America

## Abstract

Adenoviruses are DNA viruses that naturally infect many vertebrates, including humans and monkeys, and cause a wide range of clinical illnesses in humans. Infection from individual strains has conventionally been thought to be species-specific. Here we applied the Virochip, a pan-viral microarray, to identify a novel adenovirus (TMAdV, titi monkey adenovirus) as the cause of a deadly outbreak in a closed colony of New World monkeys (titi monkeys; *Callicebus cupreus*) at the California National Primate Research Center (CNPRC). Among 65 titi monkeys housed in a building, 23 (34%) developed upper respiratory symptoms that progressed to fulminant pneumonia and hepatitis, and 19 of 23 monkeys, or 83% of those infected, died or were humanely euthanized. Whole-genome sequencing of TMAdV revealed that this adenovirus is a new species and highly divergent, sharing <57% pairwise nucleotide identity with other adenoviruses. Cultivation of TMAdV was successful in a human A549 lung adenocarcinoma cell line, but not in primary or established monkey kidney cells. At the onset of the outbreak, the researcher in closest contact with the monkeys developed an acute respiratory illness, with symptoms persisting for 4 weeks, and had a convalescent serum sample seropositive for TMAdV. A clinically ill family member, despite having no contact with the CNPRC, also tested positive, and screening of a set of 81 random adult blood donors from the Western United States detected TMAdV-specific neutralizing antibodies in 2 individuals (2/81, or 2.5%). These findings raise the possibility of zoonotic infection by TMAdV and human-to-human transmission of the virus in the population. Given the unusually high case fatality rate from the outbreak (83%), it is unlikely that titi monkeys are the native host species for TMAdV, and the natural reservoir of the virus is still unknown. The discovery of TMAdV, a novel adenovirus with the capacity to infect both monkeys and humans, suggests that adenoviruses should be monitored closely as potential causes of cross-species outbreaks.

## Author Summary

Infection from adenoviruses, viruses that cause a variety of illnesses in humans, monkeys, and other animals, has conventionally been thought to be species-specific. We used the Virochip, a microarray designed to detect all viruses, to identify a new species of adenovirus (TMAdV, or titi monkey adenovirus) that caused a deadly outbreak in a colony of New World titi monkeys at the California National Primate Research Center (CNPRC), and also infected a human researcher. One-third of the monkeys developed pneumonia and liver inflammation, and 19 of 23 monkeys died or were humanely euthanized. The unusually high death rate (83%) makes titi monkeys unlikely to be natural hosts for TMAdV, and the genomic sequence of TMAdV revealed that it is very different from any other known adenovirus. The researcher developed an acute respiratory illness at the onset of the outbreak, and was found to be infected by TMAdV by subsequent antibody testing. A clinically ill family member with no prior contact with the CNPRC also tested positive. Further investigation is needed to identify whether TMAdV originated from humans, monkeys, or another animal. The discovery of TMAdV suggests that adenoviruses should be monitored closely as potential causes of cross-species outbreaks.

## Introduction

Adenoviruses, first isolated in the 1950s from explanted adenoid tissue, are double-stranded nonenveloped DNA viruses that naturally infect many vertebrates, including humans and nonhuman primates. The human adenoviruses in the *Mastadenovirus* genus, comprised of all mammalian adenoviruses, are classified into 7 species A-G, and at least 51 different serotypes (and 5 proposed types, HAdV-52 to HAdV-56) have been described to date [1], [2]. Adenoviruses are the cause of an estimated 5–10% of febrile illnesses in children worldwide [3]. Some serotypes, such as human adenovirus type 14 (HAdV-14), have been associated with severe and potentially fatal outbreaks of pneumonia in residential facilities and military bases [4]. Adenoviruses have also been associated with other clinical syndromes including conjunctivitis, hepatitis, and diarrhea [5]. In nonhuman primates, most epidemiologic studies of adenoviruses have focused on their identification in fecal samples from asymptomatic animals [6], [7], [8]. Overt respiratory disease associated with simian adenoviruses has also been observed [9]. Although adenoviruses are significant pathogens, genetically modified strains are being actively explored as potential vectors for vaccines and gene therapy [10].

Infection by adenoviruses has generally been thought to be species-specific. Human adenoviruses do not usually replicate in monkey cells in the absence of helper viruses [11], and do not productively infect rodents (and vice versa) [12]. Studies of sera from animal handlers and zoo workers exposed to chimpanzees in captivity fail to detect antibodies to chimpanzee adenoviruses [13], [14]. However, recent serological surveys have found antibodies to New World and Old World monkey adenoviruses in donor human sera from regions where the monkeys are endemic [14], [15]. In addition, phylogenetic analyses of adenoviruses from greater apes reveal that they fall precisely into “human” adenoviral species B, C, and E [7]. The high degree of sequence relatedness within members of each species suggests that at least some adenoviral strains may be capable of infecting both nonhuman primates and humans.

Beginning in May of 2009, a deadly outbreak of fulminant pneumonia and hepatitis occurred in a closed colony of New World titi monkeys of the *Callicebus* genus at the California National Primate Research Center (CNPRC). Routine microbiological testing for an infectious etiology was negative. We previously developed the Virochip (University of California, San Francisco) as a broad-spectrum surveillance assay for identifying viral causes of unknown acute and chronic illnesses [16], [17], [18], [19], [20], [21], [22]. The Virochip, a pan-viral microarray containing ∼19,000 probes derived from all viral species in GenBank (n∼2500) [21], [23], has been previously successful in detection of novel outbreak viruses such as the SARS coronavirus [22], [24] and the 2009 pandemic H1N1 influenza virus [23]. Here we apply the Virochip to identify a novel and highly divergent adenovirus as the cause of the titi monkey outbreak. In addition, we present clinical and serological evidence that this virus may have infected a researcher at the CNPRC and a family member, thus demonstrating for the first time the potential for cross-species infection by adenoviruses.

## Results

### An outbreak of fulminant pneumonia in a titi monkey colony

**Figure 1:**
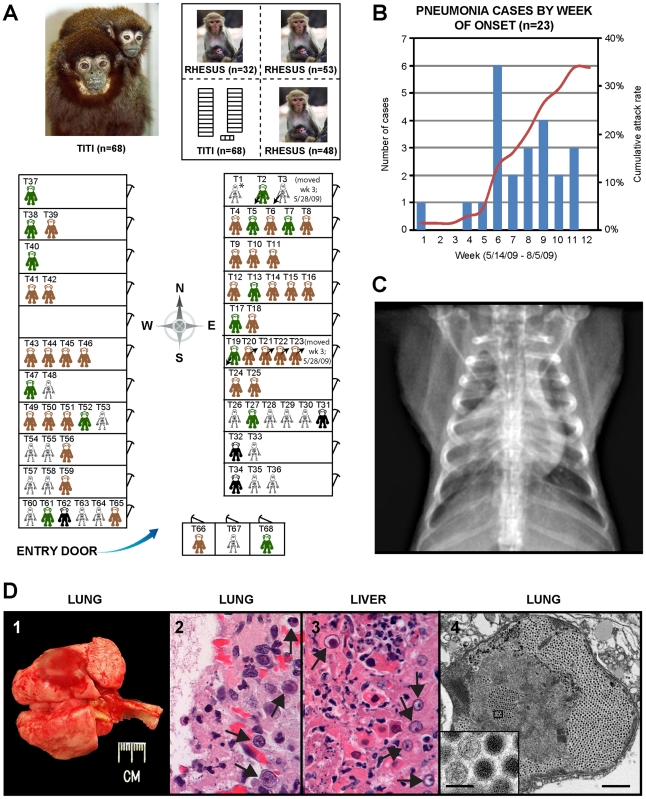
Clinical and epidemiologic features of the titi monkey outbreak. (**A**) Map of the titi monkey cages situated in one quadrant of a building, showing the locations of asymptomatic, at-risk monkeys (brown or green), affected surviving monkeys (black), and monkeys who died from their illness (skeleton). 3 monkeys were moved into the building (arrows pointing down and to the left) and 4 monkeys out of the building (arrows pointing up and to the right) during the 3^rd^ week of the outbreak. The upper left photograph shows an image of an adult male titi monkey and his infant. The upper right inset shows the location of the titi monkey cages relative to other rhesus monkey cages in the building. Asymptomatic monkeys with positive serum antibody titers to TMAdV 4 months after the outbreak are shown in green. (**B**) Epidemic curve of the outbreak, with the number of cases in blue and cumulative attack rate in red. (**C**) Anteroposterior chest radiograph of an affected titi monkey, showing bilateral basilar infiltrates and a prominent right middle lobe consolidation. (**D**) ***1*** – gross photograph of lungs at necropsy; the lungs failed to fully collapse upon opening the chest, and a single ∼1.5 cm focus of dark red discoloration (hemorrhage) can be seen in the left caudal lobe. ***2*** – photomicrograph of H&E stained lung tissue showing a severe diffuse necrotizing bronchopneumonia characterized by the presence of hemorrhage and intranuclear inclusions (arrows). ***3*** – photomicrograph of H&E stained liver tissue showing a multifocal necrotizing hepatitis with numerous intranuclear inclusions (arrows). ***4*** – transmission electron micrograph of an affected lung alveolus (scale bar  = 1 µm) filled with adenovirus-like particles (inset, scale bar  = 0.1 µm).

In early 2009, the CNPRC housed 65 titi monkeys in one quadrant of an animal building. The index case, a healthy adult titi monkey, presented on May 14, 2009 with cough, lethargy, and decreased appetite (Fig. 1A, T1). Despite aggressive treatment with intravenous fluids and antibiotics, the animal developed severe respiratory distress and was humanely euthanized 5 days later. A second case presented 4 weeks later near the entrance to the building (Fig. 1A, T54). In the interim period, 3 healthy titi monkeys had been relocated from a separate building (Fig. 1A, T2, T3, and T19), with 2 of the 3 monkeys placed into the cage formerly occupied by the index case, reflecting a total at-risk population of 68. Over the ensuing 2 months, 21 additional monkeys, including one of the relocated monkeys, presented with clinical signs similar to those shown by the index case (attack rate  = 23/68, or 34%) (Figs. 1A and 1B). Clinical signs in affected animals included cough, lethargy, poor appetite, tachypnea, and abdominal breathing. These symptoms progressed to overt respiratory distress and death or humane euthanasia within an average of 8 days. Chest radiographs typically revealed diffuse interstitial pulmonary changes and bronchoalveolar consolidation indicative of pneumonia, with right middle lobe predominance (Fig. 1C). Animals displaying clinical signs were quarantined and aggressively treated by veterinarians with supplemental oxygen, anti-inflammatory medications, bronchodilators (nebulized albuterol), broad-spectrum antibiotics, and antivirals (oseltamivir and/or ribavirin). In total, 19 animals died or were euthanized due to the illness during the outbreak (case fatality rate  = 19/23, or 83%). Only 4 monkeys survived, even though the majority of sick animals (17/23, or 74%) consisted of apparently healthy adults and juveniles. Interestingly, none of the 133 rhesus macaques (*Macaca mulatta*) housed in the same building became sick during the outbreak, and neither did any of the Old World monkeys from surrounding outdoor colonies of rhesus and cynomolgus macaques (*Macaca fascicularis*).

**Figure 2:**
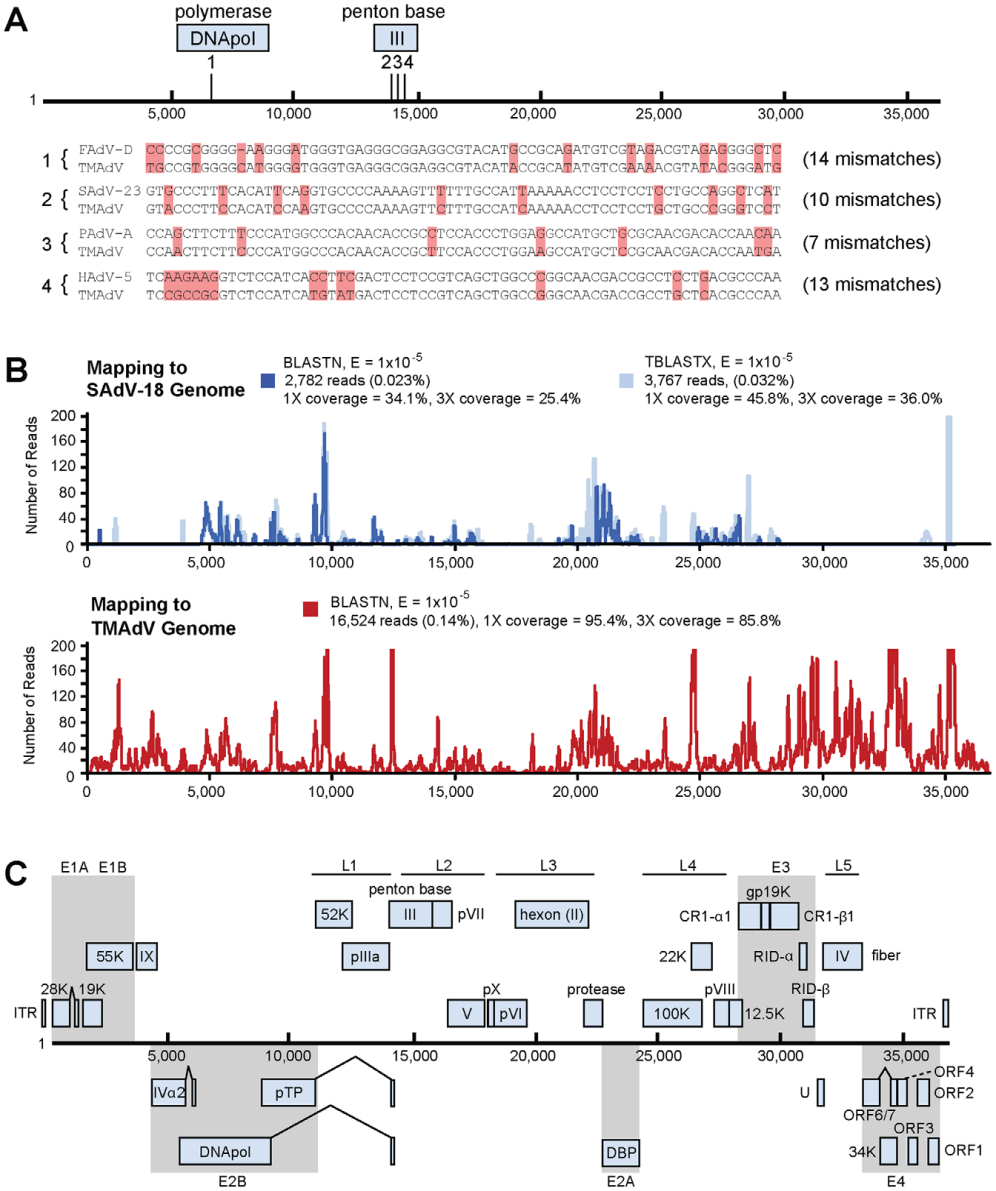
Discovery and whole-genome characterization of the novel adenovirus TMAdV. (**A**) The locations of the 4 Virochip probes derived from adenovirus sequences and used to detect TMAdV are mapped onto the ∼37 kB genome. The 4 Virochip probe sequences are also aligned with the corresponding sequence in the TMAdV genome, with mismatches highlighted in pink. (**B**) Coverage map of deep sequencing reads corresponding to TMAdV using BLASTN (blue) and TBLASTX (transparent blue) alignments to SAdV-18. The actual coverage achieved by deep sequencing as determined by alignments to the fully sequenced genome of TMAdV is much higher (red). (**C**) Genome organization of TMAdV. Predicted protein coding regions are shown as boxes. Boxes above the central black line represent open reading frames (ORFs) that are encoded on the forward strand, while boxes underneath the black line represent reverse-strand encoded ORFs. Early region ORFs are shaded in gray. The x-axis refers to the nucleotide position along the ∼37k genome of TMAdV. Abbreviations: FAdV, fowl adenovirus; SAdV, simian adenovirus; PAdV, porcine adenovirus; HAdV, human adenovirus, TMAdV, titi monkey adenovirus.

**Figure 3:**
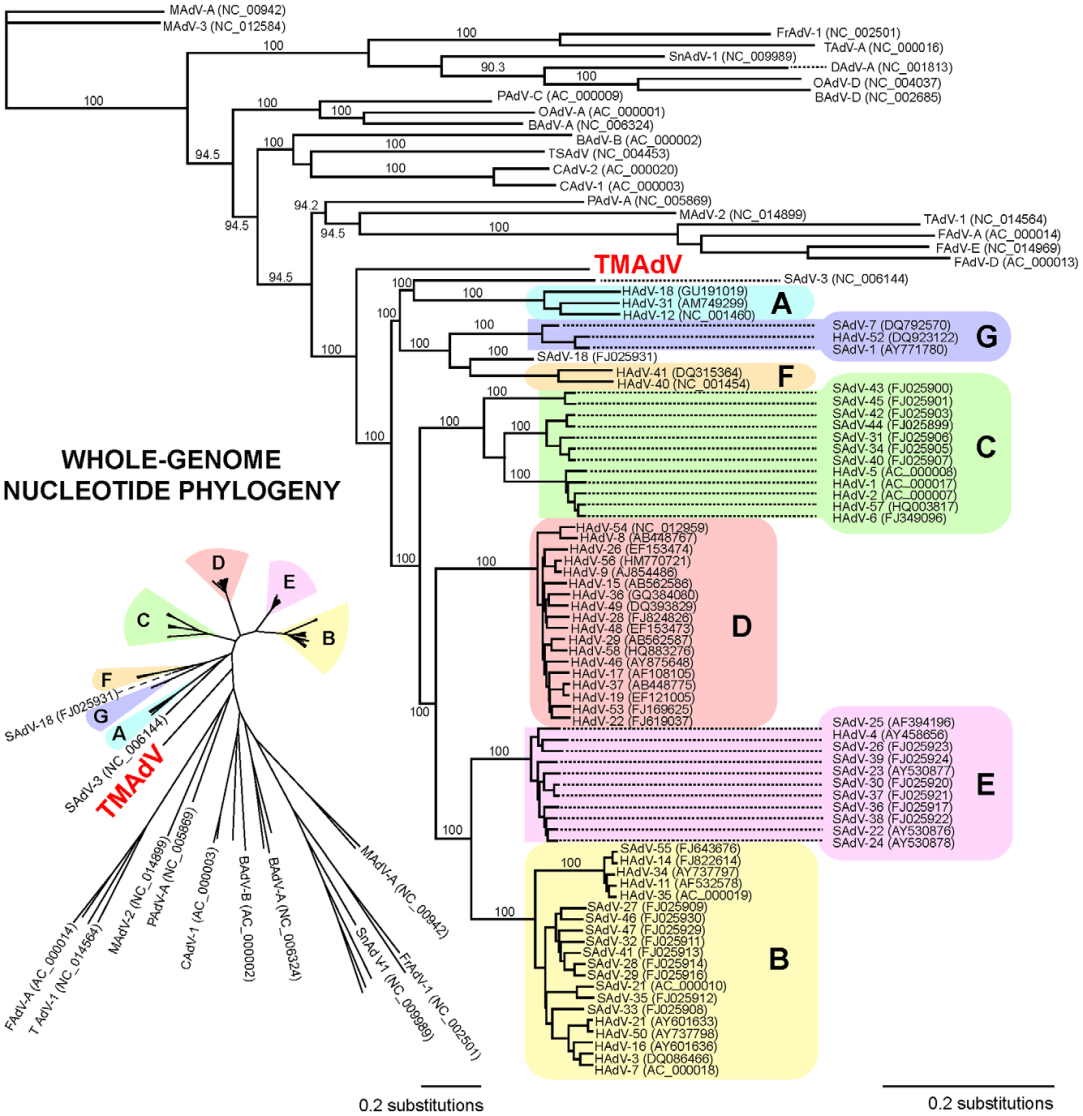
Whole-genome phylogenetic analysis of TMAdV. The whole-genome nucleotide phylogenetic tree is reconstructed from a multiple sequence alignment of all 95 unique, fully-sequenced adenovirus genomes in GenBank and TMAdV. Both rectangular cladogram and radial tree layouts are displayed. The branch corresponding to TMAdV is highlighted in boldface red. Abbreviations: HAdV, human adenovirus; SAdV, simian adenovirus; MAdV, mouse adenovirus, FrAdV, frog adenovirus; TAdV, turkey adenovirus; SnAdV, snake adenovirus; DAdV, duck adenovirus; OAdV, ovine adenovirus; BAdV, bovine adenovirus; PAdV, porcine adenovirus; TSAdV, tree shrew adenovirus; CAdV, canine adenovirus.

Gross necropsy findings were similar in all titi monkeys and were characterized primarily by diffuse, consolidated pneumonias, with occasional evidence of fibrinous pleuritis, pericardial/pleural edema, and hemorrhage (Fig. 1D-1). Some livers, spleens, and lymph nodes were found to be abnormally enlarged. Hepatic necrosis and hemorrhage, along with ascites, were occasionally appreciated. On histologic examination, the normal cellular architecture of the lung and trachea was destroyed, and prominent intranuclear inclusion bodies were observed in the liver, lung, and trachea (Figs. 1D-2 and 1D-3).

A routine microbiological workup for infectious causes of the outbreak, including bacterial, mycoplasma, and fungal cultures, was negative. Respiratory viral testing failed to detect evidence of respiratory syncytial virus, adenovirus, influenza virus A and B, human metapneumovirus, and parainfluenza virus types 1, 2, and 3.

### Virochip identification, PCR screening and electron microscopic (EM) confirmation of TMAdV

Given the clinical presentation of a severe acute viral respiratory illness and the appearance of intranuclear inclusion bodies on histological examination, we strongly suspected that a virus that had eluded detection by conventional assays was the cause of the titi monkey outbreak. Nasal, lung, and liver swab samples collected during necropsy were analyzed using the Virochip [21], [23]. Microarrays were analyzed using ranked Z-scores to assess the highest-intensity viral probes [18]. From a lung swab sample from an affected monkey, 4 of the top 80 probes on the Virochip corresponded to adenoviruses. Other viruses or viral families with ≥4 probes among the top 80, including chimpanzee herpesvirus (*Herpesviridae*), bovine viral diarrhea virus (*Flaviviridae*), and endogenous retroviruses (*Retroviridae*), were regarded as less likely to cause fulminant pneumonia and hepatitis, so were not pursued any further. The 4 adenovirus probes mapped to 2 different gene regions corresponding to the DNA polymerase and penton base (Fig. 2A). Interestingly, the 4 viral probes were derived from 2 different *Adenoviridae* genera (SAdV-23, simian adenovirus 23, PAdV-A, porcine adenovirus A, and HAdV-5, human adenovirus 5, in the *Mastadenovirus* genus; FAdV-D, fowl adenovirus D, in the *Aviadenovirus* genus), suggesting the presence of a divergent adenovirus that was not a member of any previously known species.

**Figure 4:**
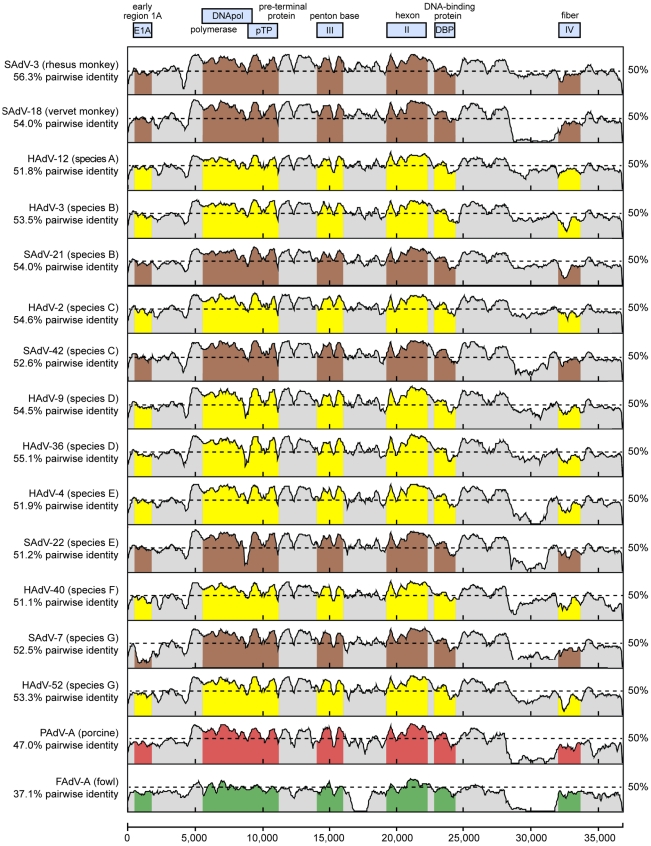
Scanning pairwise alignment of representative adenoviruses with TMAdV. The scanning nucleotide pairwise identities of TMAdV relative to representative human (yellow) or simian (brown) adenoviruses in species A–G, porcine adenovirus (red), and fowl adenovirus (green) are shown. The window size is 400 bp with a step size of 40 bp. The x-axis refers to the nucleotide position along the ∼37 k genome of TMAdV. Abbreviations: HAdV, human adenovirus; SAdV, simian adenovirus; PAdV, porcine adenovirus; FAdV, fowl adenovirus.

To confirm the Virochip finding of an adenovirus, we used consensus primers to amplify a 301 bp fragment from the hexon gene by PCR [25]. The fragment shared ∼86% nucleotide identity with its closest phylogenetic relatives in GenBank, SAdV-18, an Old World vervet monkey adenovirus, and the human species D adenoviruses. The newly identified adenovirus was designated TMAdV, or titi monkey adenovirus. Specific PCR for TMAdV was then used to screen body fluids and tissues from affected monkeys (Table 1). PCR results were positive from post-necropsy liver and lung tissues as well as from sera, conjunctival swabs, oral swabs, and nasal swabs collected at time of quarantine in 8 different affected monkeys, but were negative from a throat swab from an asymptomatic animal whose other 5 cage mates had become sick. In addition, nasal swabs were negative in 3 asymptomatic, minimal-risk titi monkeys housed in a separate building. To confirm the presence of virus in diseased tissues, we examined lung tissue from affected monkeys by transmission electron microscopy, revealing abundant icosahedral particles characteristic of adenovirus filling the alveoli (Fig. 1D-4).

**Table 1:** PCR screening for TMAdV.

Sample	Sample Type	PCR Result	Date Presenting with Clinical Signs	Date of Necropsy
***Affected, at-risk titi monkeys (died)***				
T1	serum§	−	5/14/2009	5/19/2009
T26	serum¶	+	7/23/2009	7/30/2009
T28	conjunctival swab¶	+	7/16/2009	7/25/2009
	nasal swab¶	+		
	liver swab¶	+		
	lung swab¶	+		
T29	serum¶	+	7/26/2009	7/31/2009
T30	serum¶	−	7/25/2009	7/30/3009
T33	lung swab¶	+	6/23/2009	6/29/2009
	nasal swab¶	+		
T36	lung swab¶	+	7/7/2009	7/14/2009
	lung swab¶	+		
T60	serum¶	−	7/15/2009	7/22/2009
T63	serum¶	−	6/20/2009	8/1/2009
T67	nasal swab#	−	7/7/2009	8/13/2009
	nasal swab¶	+		
***Affected, at-risk titi monkeys (survived)***				
T31	serum*	−	7/10/2009	N/A
T32	serum*	−	7/12/2009	N/A
T34	serum*	−	6/23/2009	N/A
T62	serum*	−	7/8/2009	N/A
***Asymptomatic at-risk and minimal-risk titi monkeys***				
T27	throat swab (n = 1)¶	−	N/A	N/A
at-risk titi	stool from cages (n = 14)*	−	N/A	N/A
at-risk titi	serum (n = 29)*	−	N/A	N/A
minimal-risk titi	oral swab (n = 3)¶	−	N/A	N/A
minimal-risk titi	stool from cages (n = 5)*	−	N/A	N/A
minimal-risk titi	serum (n = 8)*	−	N/A	N/A
minimal-risk titi	stool from cages (n = 8)*	−	N/A	N/A
minimal-risk titi	serum (n = 8)*	−	N/A	N/A
***Other***				
rhesus	rectal swabs (n = 26)*	−	N/A	N/A
human	serum (n = 15)∞	−	N/A	N/A
rodent	droppings (n = 2)*	−	N/A	N/A

For titi monkey cage designations (TXX), please refer to Fig. 1.

**§:** initial case.

# collected prior to outbreak.

**¶:** collected during outbreak.

*collected 2 months after outbreak.

**∞:** collected 4 months after outbreak.

Next, to assess persistent subclinical infection from TMAdV, we analyzed serum samples from at-risk asymptomatic or affected surviving monkeys 2 months after the outbreak (n = 41). All post-outbreak serum samples were negative for TMAdV by PCR (Table 1). To assess potential TMAdV shedding, stool samples collected from all cages housing titi monkeys 2 months post-outbreak were analyzed by PCR (n = 27), and were found to be negative. In addition, we checked for TMAdV in rectal swab samples from rhesus macaques housed in the same building as the titi monkeys (n = 26) and in pooled droppings from wild rodents (n = 2) living near the titi monkey cages. All macaque and rodent fecal samples were negative for TMAdV by PCR.

We also sought to determine whether PCR assays commonly used to detect human adenoviruses in clinical or public health settings could detect TMAdV. Adenovirus PCR was performed on a TMAdV-positive clinical sample, a TMAdV culture, and a human adenovirus B culture (as a positive control) using an additional 5 pairs of primers, according to previously published protocols [26], [27], [28] Three of the 5 primer pairs, designed to detect human respiratory adenoviruses B, C, and E, failed to amplify TMAdV [27]. The remaining 2 pairs of primers, both derived from highly conserved sequences in the hexon gene [26], [28], were able to detect TMAdV in culture as well as directly from clinical material.

### Whole-genome sequencing, features, and phylogenetic analysis of TMAdV

To facilitate whole-genome sequencing of TMAdV, deep sequencing of a lung swab from one affected titi monkey and lung tissue from another affected monkey was performed. Out of ∼11.9 million high-quality reads, 2,782 reads and 3,767 reads aligned to the SAdV-18 genome by BLASTN (Fig. 2B, blue) and TBLASTX (Fig. 2B, transparent blue), respectively, with reads mapping to sites across the genome. *De novo* assembly of the complete TMAdV genome from reads that aligned to SAdV-18 was not possible due to insufficient sequence coverage (<46%). The poor apparent coverage was the result of high sequence divergence of the TMAdV genome from SAdV-18, which hindered the identification of most of the 16,524 actual deep sequencing reads derived from TMAdV (Fig. 2B, red). Thus, after partial assembly of TMAdV using overlapping reads aligning to the SAdV-18 genome, remaining gaps were closed by specific PCR. The complete genome of TMAdV was found to be 36,842 base pairs in length, with a base composition of 20.8% A, 29.8% C, 29.8% G, and 19.6% T, and a GC content of 59.6%, comparable to that of adenoviral species Groups C, D, and E in the *Mastadenovirus* genus. The deduced genomic structure of TMAdV was also similar to that of other mastadenoviruses and consists of 34 open reading frames (Fig. 2C).

Whole-genome phylogenetic analysis placed TMAdV in an independent species group separate from the known human adenoviral species A–G (Fig. 3). Among all 95 fully-sequenced adenovirus genomes in GenBank, the closest simian adenoviral relatives to TMAdV were SAdV-3, SAdV-18, and SAdV-21, with pairwise nucleotide identities ranging from 54.0% to 56.3% (Fig. 4). The closest human adenoviral relatives were the species D adenoviruses, which share 54.3% to 55.1% identity to TMAdV, with human adenoviruses of other species slightly less similar (51.1%–54.6%). The placement of TMAdV into a separate group by phylogenetic analysis was also observed when looking individually at the hexon, polymerase, penton base, and fiber genes (Fig. S1). Scanning nucleotide pairwise identity plots revealed that, among the major adenovirus genes, the DNA polymerase and hexon are more conserved, whereas the E1A and fiber are more divergent (Fig. 4). The significant overall sequence divergence of TMAdV from known human and simian adenoviruses is highlighted by the finding that PAdV-A (porcine adenovirus A), a non- primate mammalian adenovirus, shared only a slightly less similar whole-genome pairwise identity to TMAdV of 47.0% (Fig. 4). In fact, in the DNA polymerase gene, TMAdV shared a pairwise identity with PAdV-A of 67.2%, comparable to its pairwise identities with the other human adenoviruses, 59%–71.7% (Figs. 4 and S1). Although TMAdV was found to be highly divergent from other adenoviruses, different isolates of TMAdV from 3 affected titi monkeys were remarkably conserved, sharing 100% identity across the full-length hexon gene (data not shown).

The high level of sequence divergence in TMAdV held true at the amino acid level as well, with amino acid identities relative to other mastadenoviruses ranging from 20.8% to 27.5% for the fiber, the most divergent protein, to 68.7%–78.2% for the hexon (Table 2). Although bearing low sequence similarity to other adenoviruses, the penton base of TMAdV contained an RGD motif that presumably binds α_v_ integrins. By both nucleotide and amino acid comparisons, the closest phylogenetic relative to TMAdV in GenBank overall was SAdV-3 (Fig. 4; Table 2). Bootscanning analysis revealed no evidence for recombination of TMAdV with other adenoviruses at either the whole-genome or individual gene level (Fig. S2).

**Table 2:** Amino acid identity of TMAdV relative to other adenoviruses.

	fiber (IV)	E1A	DBP	DNApol	penton base (III)	pTP	hexon (II) whole	hexon (II) ε:L1	hexon (II) ε:L2
SAdV-3 (rhesus monkey)	26.6%	29.9%	37.9%	61.8%	68.2%*	70.6%*	78.2%*	44.8%*	67.0%*
SAdV-18 (vervet monkey)	26.0%	30.7%	39.1%	63.2%	66.5%	68.3%	76.7%	39.1%	63.9%
HAdV-12 (species A)	26.4%	31.0%	38.2%	60.4%	64.3%	67.6%	76.2%	40.1%	64.9%
HAdV-3 (species B)	22.3%	31.4%	36.5%	61.7%	65.8%	68.8%	73.7%	38.3%	63.9%
SAdV-21 (species B)	22.2%	30.6%	36.0%	62.1%	66.1%	68.8%	72.2%	34.1%	61.2%
HAdV-2 (species C)	25.0%	32.6%*	39.5%	62.5%	67.0%	68.0%	71.4%	39.1%	61.4%
SAdV-42 (species C)	26.4%	30.5%	38.8%	62.5%	66.5%	68.1%	72.4%	37.4%	63.3%
HAdV-9 (species D)	21.9%	28.4%	38.3%	63.4%	66.1%	68.3%	74.0%	33.3%	60.0%
HAdV-36 (species D)	20.8%	30.0%	38.1%	63.3%	65.9%	68.3%	73.6%	33.1%	61.9%
HAdV-4 (species E)	26.3%	32.6%*	37.1%	62.8%	67.9%	69.8%	72.7%	30.6%	63.3%
SAdV-22 (species E)	27.5%*	31.2%	36.8%	62.9%	67.6%	70.4%	74.2%	38.6%	63.9%
HAdV-40 (species F)	26.9%	31.0%	40.1%*	62.1%	64.1%	65.9%	77.1%	42.6%	66.3%
SAdV-7 (species G)	25.5%	32.6%*	34.9%	63.3%	67.0%	68.0%	76.4%	43.8%	59.6%
HAdV-52 (species G)	24.1%	30.3%	35.6%	63.8%*	67.8%	67.9%	77.0%	46.2%*	62.6%
PAdV-A (porcine)	26.4%	23.6%	37.4%	55.4%	61.7%	57.7%	68.7%	36.4%	54.4%
FAdV-A (fowl)	1.6%	N/A	25.8%	36.5%	41.6%	31.7%	47.9%	22.3%	39.0%

The amino acid sequences of selected TMAdV proteins and the epsilon determinant of the hexon (ε: L1, loop 1, and ε: L2, loop 2) are compared to the corresponding proteins from representative human, simian, porcine, and fowl adenoviruses.

*For each protein, the entry corresponding to the adenoviral species with the highest percentage identity relative to TMAdV.

The main neutralization determinant for adenoviruses, the epsilon determinant (ε), is formed by loops 1 and 2 in the hexon protein [29]. The epsilon determinant of TMAdV was significantly divergent from that of other mastadenoviruses, with amino acid identities in loop 1 varying from 30.6% to 44.8% and in loop 2 varying from 54.4% to 67.0% (Table 2). This observation suggested that cross-neutralization of TMAdV with sera reactive against other human/simian adenoviruses is unlikely.

### Cultivation of TMAdV in human and monkey cells

**Figure 5:**
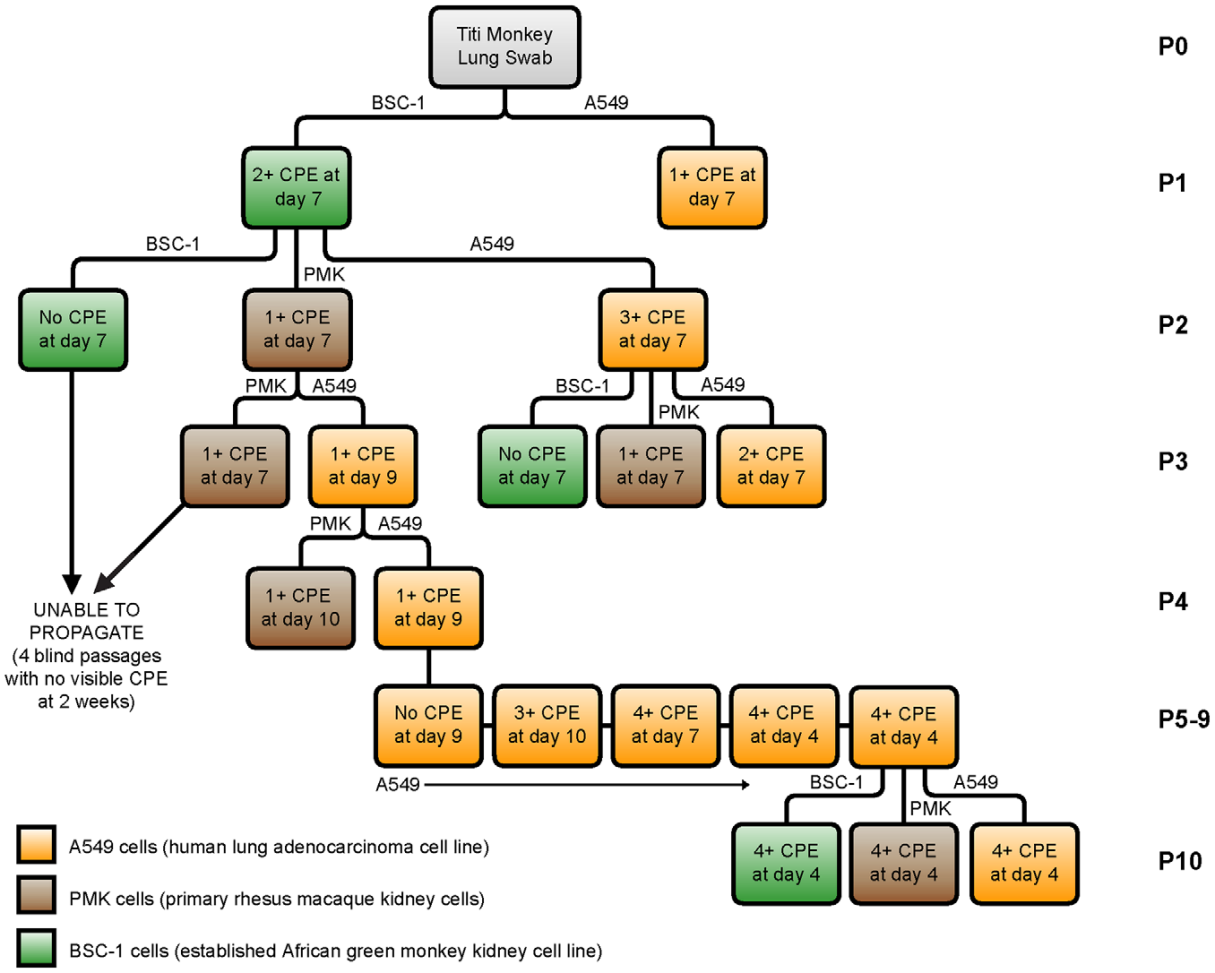
Growth and propagation of TMAdV in cell culture. The flow chart displays 10 passages (P1–P10) of TMAdV cultured in human lung adenocarcinoma (A549, orange), primary rhesus macaque kidney (PMK, brown), or established African green monkey kidney (BSC-1, green) cells.

We next attempted to culture TMAdV in an A549 (human lung adenocarcinoma) cell line, a BSC-1 (African green monkey kidney epithelial) cell line, and PMK (primary rhesus monkey kidney) cells (Fig. 5). Direct inoculation of cell cultures with a lung swab sample from an affected titi monkey produced a weak initial cytopathic effect in macaque BSC-1 and human A549 cells at day 7. However, despite multiple serial passages, we were unable to propagate the infected cell culture supernatant in either BSC-1 or PMK cells. In contrast, propagation in human A549 cells resulted in viral adaptation by passage 6 and generation of a fully adapted strain of TMAdV by passage 10 that was able to productively infect all 3 cell lines. Thus, culturing and propagation of TMAdV were successful in a human A549 cell line, but not in established or primary monkey kidney cell lines.

### An influenza-like illness in a researcher and family members during the titi monkey outbreak

In hindsight, only one individual at the CNPRC reported becoming ill during the titi monkey outbreak, the researcher in closest, daily contact with the animals. Symptoms began near the onset of the outbreak, although whether they began prior to or after identification of the index case is unclear. The researcher, with a past medical history of multiple sclerosis, initially developed symptoms of a viral upper respiratory infection (URI), including fever, chills, headache, and sore throat, followed by a dry cough and “burning sensation in the lungs” that was exacerbated by a deep breath or coughing. The researcher endorsed a history of recurrent upper respiratory infections, and did not regard the illness as related to the titi monkey outbreak. Although symptoms persisted for 4 weeks, at no time did the researcher seek medical care, and no antibiotics were taken during the illness.

We carried out contact tracing to identify family members and other individuals in close contact with the researcher. Interestingly, two family members in the household also developed flu-like symptoms about 1–2 weeks after the researcher initially became sick. Their symptoms – fever, cough and muscle aches – appeared milder than those of the researcher and completely resolved within 2 weeks. Neither individual sought medical care for these symptoms, and notably, neither had ever visited the CNPRC.

### Seroprevalence of TMAdV in monkeys and humans

**Figure 6:**
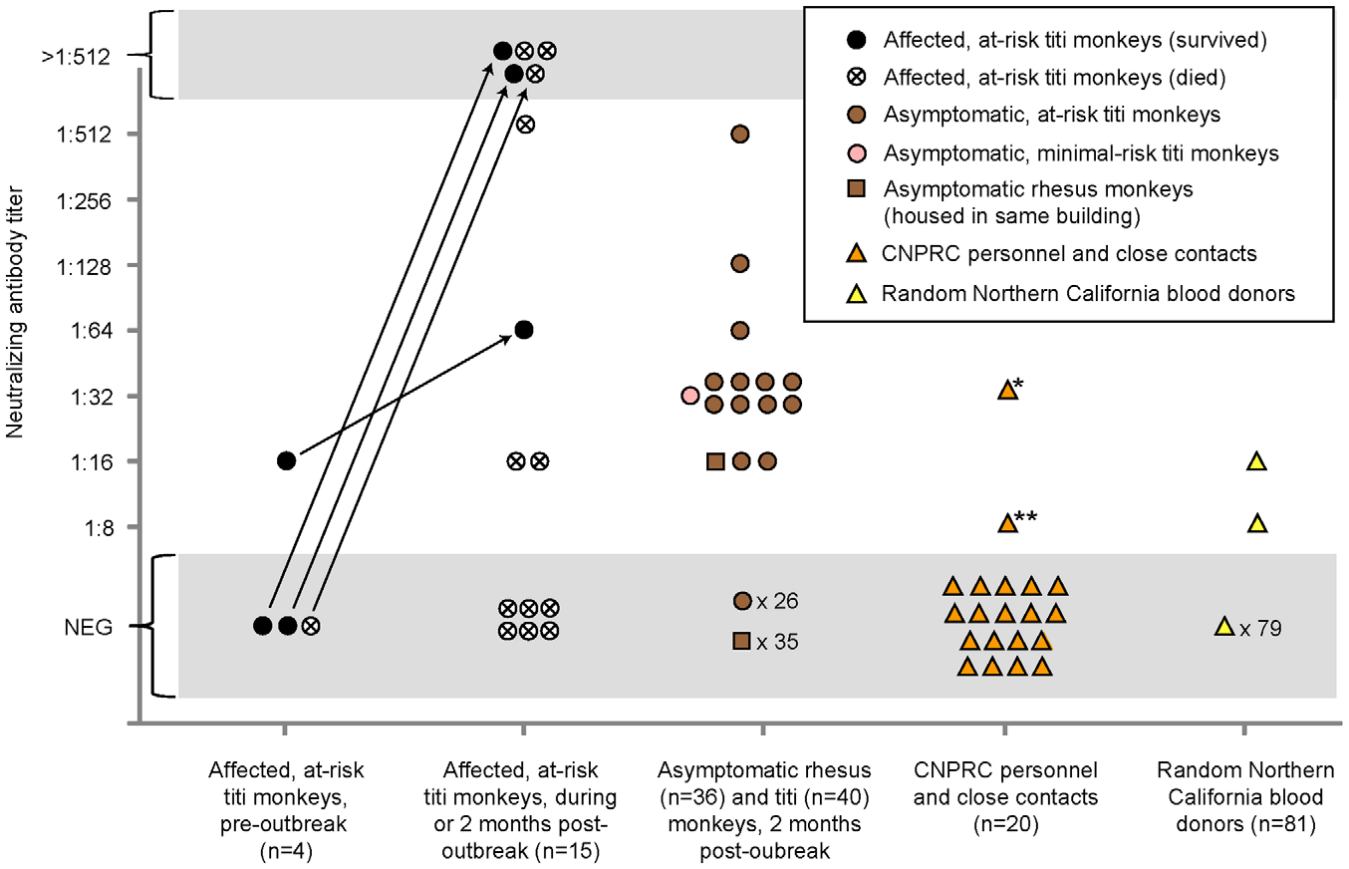
Seroprevalence of TMAdV in humans and monkeys. Sera from titi monkeys (circles), rhesus macaques (squares), and humans (triangles) were tested for antibodies to TMAdV by virus neutralization. Arrows designate pre-outbreak and post-outbreak serum samples from the same individual monkey. Pre-outbreak serum samples were previously banked in 2007. Sera from CNPRC personnel and close contacts (orange triangles) were collected 4 months post-outbreak, except for the two family members of the clinically ill researcher, whose sera were collected 1 year post-outbreak. *, clinically ill researcher; **, family member of the researcher, who was also sick. Abbreviations: CNPRC, California National Primate Research Center; NEG, negative.

To explore a potential link between the outbreak and associated illness in humans, we blindly tested available sera from titi monkeys (n = 59), rhesus macaques housed in the same building (n = 36), CNPRC personnel and close contacts (n = 20), and random human blood donors (n = 81) for evidence of recent or prior infection by TMAdV by virus neutralization (Fig. 6). Nineteen serum samples from 15 at-risk affected (symptomatic) titi monkeys were tested. Among 3 affected titi monkeys surviving the outbreak, 2 monkeys mounted a vigorous neutralizing Ab response to TMAdV, with negative pre-outbreak Ab titers (<1∶8) but antibody titers 2 months after the outbreak of >1∶512, while 1 monkey exhibited a positive but much weaker response. Affected titi monkeys who died during the outbreak exhibited a wide range of neutralizing Ab titers, from <1∶8 to >1∶512 (those without Ab likely died before mounting a response).

To investigate the possibility of subclinical infection by TMAdV, we also examined serum samples from asymptomatic titi monkeys (n = 40) and nearby rhesus macaques (n = 36), collected 2 months after the outbreak. Fourteen of 40 asymptomatic titi monkeys tested (35%) had antibody to TMAdV, indicating that the incidence of subclinical infection was significant (Fig. 1A; Fig 6). In fact, one of the 14 asymptomatic titi monkeys with positive Ab titers was located in the minimal-risk building. In contrast, only 1 of 36 rhesus macaque samples was positive, with an Ab titer of 1∶16. The 1 antibody-positive rhesus serum sample was negative by specific PCR for TMAdV (data not shown), as was stool from the cage in which the rhesus monkey was housed (Table 1).

Approximately 4 months after the outbreak, serum samples were collected from CNPRC personnel in direct contact with the titi monkeys. Serum samples were also collected from the two family members of the clinically ill CNPRC researcher 1 year after the outbreak. Only two samples were found positive for neutralizing Abs to TMAdV: (1) Ab titers for the clinically ill researcher were 1∶32, and (2) Ab titers for one of the family members of the clinically ill researcher were 1∶8.

Among 81 random blood donors from the Western United States, 2 individuals (2/81, 2.5%) had positive Ab titers of 1∶16 and 1∶8. Pooled rabbit sera containing antibodies to human adenovirus serotypes 1 through 35, representing species A–E, were unable to neutralize TMAdV (data not shown). Thus, the results of our serological survey appear unlikely to be due to nonspecific cross-reactivity from prior exposure to known human adenoviruses.

## Discussion

In this study, we employed a pan-viral microarray assay, the Virochip, to identify a novel adenovirus associated with a fulminant pneumonia outbreak in a colony of New World titi monkeys. Despite the absence of an animal model, which precludes a strict fulfillment of Koch's postulates, there are several lines of evidence implicating this novel adenovirus, TMAdV, as the cause of the outbreak. First, conventional testing for other pathogens, including other viruses by Virochip, was negative, and affected monkeys did not respond to empiric therapy with antibiotics or antivirals (ribavirin and oseltamivir in anecdotal use are not effective against adenoviral infections) [30]. Second, the clinical presentation of pneumonia and hepatitis is consistent with the known spectrum of disease associated with adenoviral infections. Third, TMAdV sequence was recovered by PCR in various body fluids and tissues from affected monkeys, including blood, respiratory secretions, and lung/liver tissue (Table 1). Fourth, the finding of intranuclear inclusions in diseased tissues, as well as direct visualization of adenoviral-like particles (TMAdV) in lung alveoli by electron microscopy (Figs. 1D-2 to 1D-4), support a primary role for TMAdV in the pathogenesis of tissue injury in affected monkeys. Finally, there was a significant neutralizing Ab response in surviving animals, with 2 monkeys having titers undetectable prior to the outbreak but rising to >1∶512 at convalescence (Fig. 6).

Although TMAdV retains the core genomic features common to all adenoviruses (Fig. 2C), phylogenetic analysis clearly places TMAdV within a separate branch, with no closely related neighbors (Figs. 3 and S1). A phylogenetic distance of >10% combined with the lack of cross-neutralization defines TMAdV as a new species [31]. Since emerging adenovirus strains such as HAdV-14 and HAdV-D22/H8 (otherwise known as HAdV-D53) are known to arise from recombination events among related ancestral strains [32], [33], we performed bootscanning analysis to look for such events in TMAdV. The bootscanning analysis, however, failed to show evidence of recombination, likely because closely related and/or ancestral strains to TMAdV have not yet been identified.

Entry of adenoviruses into cells involves an initial attachment of the fiber knob to the cell receptor, followed by internalization via a secondary interaction of the penton base with α_v_ integrins [34], [35]. The presence of an RGD motif in the TMAdV penton base implies that the virus uses α_v_ integrins for internalization [35]. However, the high sequence divergence in the fiber protein (Table 2), as well as the absence of fiber motifs conserved among adenoviruses that bind CAR [36], [37] (coxsackievirus-adenovirus receptor) or CD46 [38], [39], [40] (data not shown), suggest that neither of these two human adenoviral receptors may be the attachment receptor for TMAdV. Further studies will be necessary to identify the preferred cellular attachment and internalization receptors for TMAdV.

Despite its isolation from affected titi monkeys, we were unable to propagate TMAdV in both established (BSC-1) and primary (PMK) monkey kidney cells (Fig. 4). The virus, however, grew efficiently in a human A549 lung adenocarcinoma cell line. One explanation for this finding is that TMAdV may be unable to productively infect cells derived from Old World monkeys (e.g. rhesus and African green monkeys). An alternative possibility is that successful propagation of TMAdV may depend on infection of a specific host cell type, such as A549 lung, and not BSC-1 or PMK kidney cells. Nevertheless, after 10 passages in human A549 cells, the fully adapted strain of TMAdV exhibits an extended host range with the ability to productively infect both monkey and human cells. This observation implies that TMAdV possesses an inherent capacity to cross the species barrier and infect both humans and nonhuman primates. Efforts to identify host range and cell tropism of TMAdV, as well as the specific sequence changes responsible for adaptation to growth in cell culture, are currently underway.

The virulence of TMAdV in healthy and apparently immunocompetent titi monkeys (83% case fatality rate) is highly unusual for infections by adenovirus. In humans, deaths due to adenovirus infections or outbreaks are generally low (up to 18% for pneumonia associated with HAdV-14 [4]). Furthermore, severe infections from human adenoviruses are more commonly associated with older age, immunosuppression, and chronic underlying conditions such as kidney failure [4], [41]. Young, healthy individuals are in general much less likely to succumb to adenoviral-related illness. The severity of TMAdV-related illness in affected titi monkeys suggests that this species of monkey may not be the natural host for the virus. The failure to detect fecal shedding of TMAdV in convalescent or asymptomatic animals also suggests that the virus does not normally infect titi monkeys (Table 1).

Although the exact origin of TMAdV remains unclear, we can speculate on several possibilities. One possibility is that a cross-species “jump” from captive macaques to a susceptible colony of titi monkeys precipitated the outbreak. As there have been no new introductions of monkeys into the closed colony for the past 2 years, this conjecture relies on asymptomatic infection and transmission of TMAdV in the captive rhesus/cynomolgus macaque population at the CNPRC. CNPRC personnel who visited macaque areas would occasionally enter titi rooms with no change in personal protective equipment, thus providing a potential route of transmission for the virus. In addition, specific antibodies were detected in 1 of 36 (2.8%) asymptomatic rhesus macaques housed in the same building (Fig. 6), indicating that TMAdV has the capacity to infect this species of Old World monkey. Notably, the closest identified phylogenetic relative to TMAdV among the complete genomic sequences available in GenBank is a rhesus monkey adenovirus, SAdV-3 (Fig. 4; Table 2). Furthermore, serological evidence for cross-species adenoviral transmission events between different nonhuman primate species has been previously reported in the literature [42].

Although we failed to detect TMAdV in rodent droppings found near titi monkey cages (Table 2), it is still possible that the virus arose from an unknown animal reservoir. In this regard, the high sequence divergence of TMAdV relative to the known human/simian adenoviruses (Fig. 3), and comparable sequence similarity in the polymerase gene to a porcine adenovirus (Figs. 3 and S1) are striking. The four-week interval between the index case and the second case appears overly long given a typical incubation period for adenovirus infections of no more than 1 week [43]. This may be explained by our finding of a high rate of subclinical infection by TMAdV in asymptomatic titi monkeys (35%), but may also be due to separate introductions of TMAdV into the colony from an as-yet unidentified reservoir.

Our study data also support the potential for cross-species transmission of TMAdV between monkeys and humans. The researcher's fever, cough, and pleuritic symptoms (“burning sensation in the lungs”) are consistent with the development of a prolonged viral respiratory illness. Interestingly, pleurisy has been specifically reported in association with certain human adenovirus infections [44]. The clinical presentation, time of illness concurrent with the onset of the outbreak, and presence of neutralizing Abs in convalescent serum all strongly point to primary infection of the researcher by TMAdV. The detection of weakly neutralizing Abs (1∶8) in a serum sample from a sick family member of the researcher also suggests that TMAdV may be capable of human-to-human transmission. The decreased levels of neutralizing Abs to TMAdV in the researcher (1∶32) and a family member (1∶8) relative to those in infected titi monkeys (up to >1∶512) are consistent with a recent study showing much higher levels of neutralizing antibodies in chimpanzees than in humans with adenovirus infections, possibly due to more robust adenovirus-specific T-cell responses in humans than in monkeys [45].

Several lines of evidence support the contention that the direction of TMAdV transmission was zoonotic (monkeys to humans) rather than anthroponotic (humans to monkeys). First, the closest known relative to TMAdV in GenBank is SAdV-3, an Old World monkey adenovirus (Fig. 3; Table 2). Second, our results show that PCR assays for human adenoviruses in common use are capable of detecting TMAdV. Although sequencing of PCR amplicons for human adenoviruses is not performed routinely in diagnostic virology, TMAdV would presumably have been detected previously in large-scale studies of hexon sequencing of Ad field isolates if it were circulating in the community [46], [47]. Finally, the available sequence data in GenBank is heavily biased towards human adenoviruses, and much less is known about the potential diversity of the simian adenoviruses. We also cannot formally exclude the possibility that the outbreak arose from anthroponotic transmission. In our study, 2 of 81, or 2.5% of random adult blood donors exhibited borderline titers of neutralizing antibody to TMAdV, indicating either a low prevalence of TMAdV in the human population or cross-reactivity to a related virus (although no evidence of cross-reactivity was found with HAdV serotypes 1 through 35). Future large-scale studies of TMAdV seroepidemiology will be needed to better understand transmission of TMAdV between monkeys and humans. Nevertheless, our discovery of TMAdV, a novel adenovirus with the capacity to cross species barriers, highlights the need to monitor adenoviruses closely for outbreak or even pandemic potential.

## Materials and Methods

### Ethics statement

This study was carried out in strict accordance with the recommendations in the Guide for the Care and Use of Laboratory Animals of the National Institutes of Health. The use and care of all animals followed policies and guidelines established by the University of California, Davis Institutional Animal Care and Use Committee (IACUC) and CNPRC (Animal Welfare Assurance #A3433-01). The protocol for the maintenance and breeding of the titi monkey colony was approved by the University of California, Davis IACUC (Protocol #15730). No specific animal research protocol was drafted for this study, as only excess clinical samples were analyzed for diagnostic purposes. Animals in extreme respiratory distress were humanely euthanized by veterinarians. Extensive veterinary care was provided to all animals affected by the outbreak in order to minimize pain and distress.

Serum samples from staff at the CNPRC, close contacts, and random adult blood donors were collected under protocols approved by institutional review boards of the University of California, Davis (Protocol #200917650-1) and University of California, San Francisco (Protocol #H49187-35245-01). Specifically, written informed consent was obtained from staff at the CNPRC and close contacts for analysis of their samples. Any potentially identifying information has been provided with the explicit permission of the individuals involved.

Sera from random blood donors were obtained from the Blood Systems Research Institute (San Francisco, CA); sera were derived from affiliated donor banks in California (Blood Centers of the Pacific, San Francisco, CA), Nevada (United Blood Service, Reno, NV), and Wyoming (United Blood Services, Cheyenne, Wyoming) and de-identified prior to analysis.

### The California National Primate Research Center (CNPRC)

The California National Primate Research Center (CNPRC), which houses over 5,000 nonhuman primates, is a part of the National Primate Research Centers Program and is accredited by the Association for the Assessment and Accreditation of Laboratory Animal Care, International (AAALAC). At the beginning of 2009, the CNPRC maintained a colony of 74 titi monkeys (*Callicebus cupreus*) and a colony of over 4,500 rhesus macaques (*Macaca mulatta*). No new animals have been introduced into either colony for over 2 years. All titi monkeys are maintained in small social groups, while rhesus macaques are maintained in small or large social groups. All animal facilities are maintained in compliance with United States Department of Agriculture specifications.

Eighty-eight percent of the titi monkey population (n = 65) were housed in 1 quadrant of an indoor animal building, and all titi monkeys demonstrating clinical signs originated from this building (i.e. “at-risk” room) (Fig. 1A). Rhesus macaques (n = 133) were housed in the other 3 quadrants of this same building, and surrounding the building were outdoor housing units with rhesus macaques and cynomolgus macaques (*Macaca fasicularis*). Three additional titi monkeys were moved into the at-risk room less than 2 weeks after presentation of the index case, reflecting a total at-risk population of 68 animals. The remaining 6 titi monkeys were housed in an indoor animal building greater than 500 yards from the at-risk population (i.e. “minimal-risk” room).

### Outbreak investigation and microbiological testing

The outbreak lasted approximately 3 months from May to August of 2009. Affected titi monkeys died from 3–24 days after appearance of clinical signs, with an average time to death or humane euthanasia of 8 days. Clinical and epidemiological data, including daily census reports, were tracked and recorded by veterinary and management staff. All personnel entering the titi monkey rooms (both at-risk rooms and minimal-risk rooms) needed to pass within approximately 20 feet of macaque enclosures prior to entry. CNPRC personal protective equipment (PPE) policy requires a change of PPE between entrance/exit of animal rooms housing different species. Staff compliance of this policy may have been compromised. Measures have since been taken by CNPRC management to ensure compliance with existing policies.

Bacterial, mycoplasma, and fungal cultures were performed at the CNPRC. Clinical samples were also sent to an outside laboratory (Focus Diagnostics, Cypress, CA) for respiratory viral testing by centrifugation-enhanced shell vial culture followed by direct fluorescent antibody staining for 8 viruses (respiratory syncytial virus, adenovirus, influenza virus A and B, parainfluenza virus types 1, 2, and 3, and human metapneumovirus).

### Gross, histopathological, and ultrastructural analyses

Gross and histopathological analyses of post-mortem tissues were performed by a board-certified veterinary pathologist specializing in nonhuman primate/laboratory animal medicine, a branch of Primate Services at the CNPRC. At necropsy, tissue samples from the trachea, lung, and liver were collected and fixed in 10% formalin. Tissues were routinely processed and embedded in paraffin. 3-µm sections were stained with hematoxylin and eosin (HE) and examined by light microscopy. For transmission electron microscopy, tissue fragments (2×2 mm) were excised from paraffin blocks of lung, deparaffinized, and processed as previously described [48].

### Nucleic acid extraction and cDNA library preparation

Total nucleic acid was extracted from body fluid or swab samples using commercially available kits (Qiagen, Valencia, CA). 200 µL of sample were passed through a 0.22 µm filter (Millipore, Temecula, CA) to remove bacteria and cellular debris and then treated with Turbo DNase (Ambion, Culver City, CA) to degrade host genomic DNA prior to extraction. For tissue samples, lung or liver tissue was homogenized in a 15 mL Eppendorf tube using a disposable microtube pestle (Eppendorf, San Diego, CA) and scalpel, and RNA extraction was then performed using TRIzol LS (Invitrogen, Carlsbad, CA), followed by isopropanol precipitation and two washes in 70% ethanol. Extracted nucleic acid was amplified using a random PCR method to generate cDNA libraries for Virochip and deep sequencing analyses as previously described [18], [21].

### Virochip analysis

The current 8×60 k Virochip microarrays used in this study contain 19,058 70mer probes representing all viral species in GenBank, and combine probes from all previous Virochip designs [17], [18], [21], [23]. Four probes derived from 2 different *Adenoviridae* genera (SAdV-23, PAdV-A, HAdV-5, and FAdV-D) yielded an adenovirus signature on the Virochip that was found to be TMAdV. With the exception of SAdV-23, these highly conserved probes are part of the core Virochip design and were derived from all available adenoviral sequences in GenBank as of 2002 [21]. One explanation why more high-intensity probes to simian adenoviruses were not seen by Virochip analysis is that the genomes of many simian Ads, including SAdV-3 and SAdV-18 (the two closest phylogenetic relatives to TMAdV in GenBank), were not sequenced until after 2004 [7], [49], and thus their genomes are not as well-represented on the Virochip microarray.

Virochip analysis was performed as previously described [21], [23]. Briefly, samples were labeled with Cy3 or Cy5 fluorescent dye, normalized to 10 pmol of incorporated dye, and hybridized overnight using the Agilent Gene Expression Hybridization kit (Agilent Technologies, Santa Clara, California). Slides were scanned at 3 µm resolution using an Agilent DNA Microarray Scanner. Virochip microarrays were analyzed with Z-score analysis [18], hierarchical cluster analysis [50], and E-Predict, an automated computational algorithm for viral species prediction from microarrays [51]. Only Z-score analysis, a method for assessing the statistical significance of individual Virochip probes, yielded a credible viral signature on the microarray.

### PCR screening

We initially used consensus primers derived from a highly conserved region of the hexon gene to confirm the Virochip finding of an adenovirus by PCR [25]. After recovering the full genome sequence, we then designed a set of specific PCR primers for detection of TMAdV, TMAdV-F (5′-GTGACGTCATAGTTGTGGTC) and TMAdV-R (5′-CTTCGAAGGCAACTACGC). The TMAdV-specific quantitative real-time PCR was performed on a Stratagene MX3005P real-time PCR system using a 25 µL master mix consisting of 18 µL of water, 2.5 µL of 10X *Taq* buffer, 1 µL of MgCl_2_ (50 mM), 0.5 µL of deoxynucleoside triophosphates (dNTPs; 12.5 mM), 0.5 µL of each primer (10 µM), 0.5 µL SYBR green, 0.5 µL of *Taq* polymerase (Invitrogen, Carlsbad, CA), and 1 µL of extracted nucleic acid. Conditions for the PCR reaction were 40 cycles of 94°C for 30 s, 55°C for 30 s, and 72°C for 30 s. Amplicons were purified on a 2% agarose gel, cloned into plasmid vectors using TOPO TA (Invitrogen, Carlsbad, CA), and sent to an outside company (Elim Biopharmaceuticals, Hayward, CA) for Sanger sequencing in both directions using vector primers M13F and M13R.

To assess linearity and limits of sensitivity for the TMAdV PCR assay, 12 serial log dilutions were made of a standard plasmid constructed by cloning the 157-bp TMAdV amplicon into a TOPO plasmid vector. Purified plasmid clones at each serial dilution were quantified using a Nanodrop spectrophotometer and then spiked into negative serum, stool, or oral swab sample matrix, each matrix consisting of a pool of 10 sera, 10 stool samples, or 3 oral swabs, respectively. For each sample type, a standard curve for the TMAdV PCR assay was calculated from 3 PCR replicates at each dilution of nucleic acid extracted from the spiked matrix (data not shown). To determine limits of sensitivity for the assay, probit analysis of results from 6 PCR replicates of 7 serial log dilutions (from a starting concentration of ∼1.2×10^5^ copies/mL) was performed using SPSS 16.0 (SPSS Inc., Chicago, IL). By probit analysis, the 95% limit of detection for TMAdV was 781, 377, or 35 viral genome equivalents/mL for serum, stool, or oral swab samples, respectively (data not shown).

### Whole-genome sequencing

To facilitate whole-genome sequencing of TMAdV, we prepared amplified cDNA/DNA libraries for deep sequencing from lung tissue and a lung swab sample from 2 different monkeys using previously published protocols [23], [52]. Briefly, randomly amplified libraries were cleaved with a Type IIs restriction endonuclease (*GsuI*) and truncated adapters were ligated on the resulting strand ends. Full-length adapters containing strict 6-nt barcodes were added via an additional 15 cycles of PCR. Amplified libraries were size-selected on a 2% agarose gel at approximately 350 bp average length and then sent to an outside company (Elim Biopharmaceuticals, Hayward, CA) for deep sequencing on an Illumina Genome Analyzer IIx (Illumina, San Diego, CA). Paired-end reads were sequenced for 73 cycles in each direction. Paired-end reads were subsequently filtered to eliminate low-complexity sequences with a Lempel-Ziv-Welch (LZW) compression ratio below 0.4 [53], split into individual reads, classified by barcode, and stripped of any remaining primer sequences using BLASTN alignments (word size = 11, E-value = 1×10^−5^). After low-complexity filtering and barcode/primer trimming, 11,950,557 sequence reads remained, with each read consisting of 67 nucleotides, for a total of ∼800 megabases of sequence. Reads were then aligned using BLASTN (word size = 11, E-value = 1×10^−5^) and TBLASTX (word size = 11, E-value = 1×10^−5^) to the genome sequence of SAdV-18 (Fig. 2B). Overlapping reads aligning to SAdV-18 were used to assemble portions of the TMAdV genome with Geneious software (version 3.6.5) [54], employing the SAdV-18 genome as a reference sequence and requiring a 20-bp minimum overlap and 95% overlap identity. Aligning reads were also used to design PCR primers to close remaining gaps in the TMAdV genome. Amplicons derived from specific TMAdV PCR primers were gel-purified, cloned, and sequenced as described above. The 5′ end corresponding to the inverted terminal repeat (ITR) of TMAdV was obtained by PCR using a forward degenerate consensus primer and a reverse TMAdV-specific primer. The 3′ end was recovered using a forward primer near the 3′ end of the genome and a reverse primer derived from 5′-ITR sequence.

### Structural features and phylogenetic analysis

To identify predicted coding regions in the TMAdV genome, we used the fully annotated genome sequence of SAdV-21 in GenBank as a reference. First, we aligned the two genomes and identified all ORFs that were present with Geneious [54]. We then selected the candidate ORF that best matched the corresponding ORF in the annotated reference genome. For adenovirus genes that are spliced (e.g. E1A), the identification of a GT-AG intron start-stop signal was used to pinpoint the correct ORF. To confirm the accuracy of the coding sequence, the sequence of each identified ORF was aligned to a database containing all adenoviral proteins in GenBank by BLASTX.

To generate whole-genome and individual gene nucleotide phylogeny trees, all 95 fully sequenced unique adenovirus genomes were first downloaded from GenBank. Multiple sequence alignments were then performed on a 48-core Linux system using ClustalW-MPI [55]. Trees were constructed after bootstrapping to 1000 replicates by the neighbor-joining method (based on Jukes-Cantor distances) in Geneious [54], [56]. Pairwise alignments were calculated using Shuffle-LAGAN (window size, 400 bp; step size 40 bp; translated anchoring), a glocal alignment algorithm that is able to calculate optimal alignments by using both local alignments and global maps of sequence rearrangements (e.g. duplications of the fiber gene in adenovirus genomes with 2 fibers) [57]. Sliding window analysis of the Shuffle-LAGAN pairwise alignments was performed using the online mVISTA platform [58]. More accurate alignments were obtained with Shuffle-LAGAN than with either ClustalW-MPI or Geneious (data not shown). Bootscanning analysis was performed according to the Kimura 2-parameter method using 1000 replicates with Simplot (version 3.5.1) [59]. Pairwise amino acid amino acid alignments between predicted TMAdV proteins and corresponding proteins in other adenoviruses (Table 2) were performed using Geneious [54].

### Virus cultivation

A549 (human lung adenocarcinoma) and BSC-1 (African green monkey kidney epithelial) cell lines as well as PMK (primary rhesus monkey kidney) cells are routinely maintained at the Viral and Rickettsial Disease Laboratory (VRDL) branch of the California Department of Public Health. Media consisting of Hank's medium (for A549 cells) or Dulbecco's modified Eagle's medium (DMEM) (for BSC-1 cells) were supplemented with 1×nonessential amino acids (Invitrogen, Carlsbad, CA), 10% fetal bovine serum, 100 U of penicillin/mL and 100 µg of streptomycin/mL. PMK cells were maintained in tubes containing growth media and antibodies to SV-40 and SV-5 polyomaviruses (Viromed, Pasadena, CA). Clinical samples were clarified by centrifugation for 10 min×4000 *g* and passaged through a 0.2-µm filter. Cell culture passages were subjected to 3 freeze-thaw cycles and clarified as above. After achieving 80–90% confluency, cell culture media were changed to maintenance media with 2% FBS and were inoculated with 200 µL of clinical sample or 100 µL of passaged viral supernatant. Viral replication was monitored over 14 days by visual inspection under light microscopy for cytopathic effect (CPE). To confirm the generation of infectious virus, viral supernatants were quantitated by an end-point dilution assay.

### Virus neutralization assay (human and monkey sera)

A virus stock of TMAdV (passage 7) was produced on human A549 cells, aliquoted, and quantitated by end-point dilution. To perform the virus neutralization assay, 55 µL of viral supernatant at a concentration of 100 TCID_50_ and 55 µL of serum (starting at a 1∶8 dilution) were mixed and incubated for 1 hour at 37°C. As a control for each serum sample, 55 µL of culture media and 55 µL of serum were mixed and treated in an identical fashion. While mixtures were incubating, A549 cells grown in T-25 plates were trypsinized and 4,000 cells in 100 µL of media were added to each well of a 96-well plate. After incubation, 100 µL of mixture were inoculated into appropriate wells containing 4,000 cells per well and the entire plate was placed in a 37°C 5% CO_2_ incubator. Cells in the plate wells were observed for evidence of CPE every other day for 1 week. For wells that showed inhibition of viral CPE, the corresponding serum samples were diluted in six 2-fold steps and then retested. The reciprocal of the highest dilution that completely inhibited viral CPE was taken as the neutralizing antibody titer.

### Virus neutralization assay (rabbit typing sera)

To assess cross-neutralization of TMAdV by known human adenoviruses, 7 pools of in-house reference sera at the VRDL (rabbit hyperimmune sera) and collectively containing antibodies to human adenovirus serotypes 1 through 35 were available for testing. For each pool, 55 µL of rabbit sera and 55 µL of viral supernatant at a concentration of 100 TCID_50_ were mixed, incubated for 1 hour at 37°C, and inoculated onto A549 cells in wells of a 96-well plate as described above. Cells in the plate wells were observed for evidence of CPE every other day for 1 week.

### Microarray and nucleotide sequence accession numbers

All Virochip microarrays used in this study have been submitted to the NCBI GEO database (study accession number GSE26898; microarray accession numbers GSM662370-GSM662391; microarray design accession number GPL11662). The annotated, whole-genome sequence of TMAdV has been submitted to GenBank (accession number HQ913600). Deep sequencing reads have been submitted to the NCBI Sequence Read Archive (accession number SRA031285).

## Supporting Information

Figure S1 **Phylogenetic analysis of the hexon, polymerase, penton base, and fiber genes of TMAdV.** A multiple sequence alignment of selected genes from all 95 unique, fully-sequenced adenovirus genomes in GenBank and TMAdV is performed and the results displayed as a radial phylogenetic tree. The branch corresponding to TMAdV is highlighted in boldface red. Abbreviations: HAdV, human adenovirus, SAdV, simian adenovirus; PAdV, porcine adenovirus; FAdV, fowl adenovirus.
(TIF)

Figure S2 **Bootscanning recombination analysis of TMAdV.** Bootscanning analysis was initially performed with all 95 unique, fully-sequenced adenovirus genomes in GenBank (data not shown). After removal of similar viral genomes, bootscan plots of the whole genome and individual genes from a subset representing human/simian adenoviruses in species A–G and all non-primate vertebrate adenoviruses were generated. The window size is 400 bp with a step size of 40 bp for the whole genome, and 200 bp with a step size of 20 bp for the individual genes. The x-axis refers to the nucleotide position. For definition of abbreviations, please refer to Fig. 3.
(TIF)
