# Correction: Cross-Species Transmission of a Novel Adenovirus Associated with a Fulminant Pneumonia Outbreak in a New World Monkey Colony

**DOI:** 10.1371/annotation/59703f7f-9506-49d1-b339-09ee31510e89

**Published:** 2011-08-25

**Authors:** Eunice C. Chen, Shigeo Yagi, Kristi R. Kelly, Sally P. Mendoza, Nicole Maninger, Ann Rosenthal, Abigail Spinner, Karen L. Bales, David P. Schnurr, Nicholas W. Lerche, Charles Y. Chiu

The legend for Figure 1 states that the arrows designate cells with “intranuclear inclusions”, whereas some of the arrows previously did not point to these cells, but rather to normal nucleoli. The arrows now correctly point to cells containing intranuclear inclusions as visualized by light microscopy. Please see the corrected Figure 1 here: 

**Figure:**
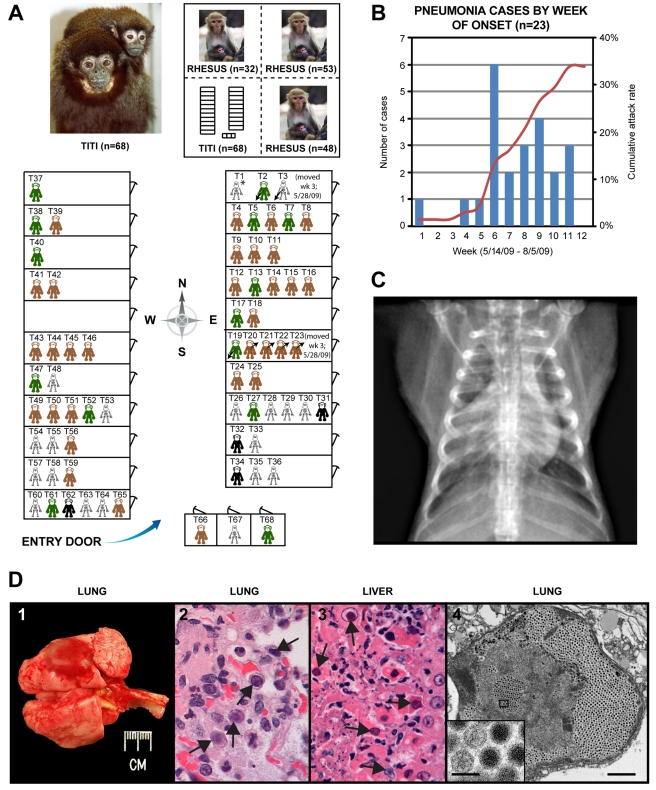


[^] [^] 

