# Correction: Cross-Species Transmission of a Novel Adenovirus Associated with a Fulminant Pneumonia Outbreak in a New World Monkey Colony

**DOI:** 10.1371/annotation/c9a506d7-e8ba-4aaf-ab04-8ed0b383f5d9

**Published:** 2011-08-25

**Authors:** Eunice C. Chen, Shigeo Yagi, Kristi R. Kelly, Sally P. Mendoza, Nicole Maninger, Ann Rosenthal, Abigail Spinner, Karen L. Bales, David P. Schnurr, Nicholas W. Lerche, Charles Y. Chiu

Dr. Don R. Canfield should be included in the author byline instead of the Acknowledgements, and Dr. Ross P. Tarara should be included in the byline as well. Dr. Tarara should be listed as the fifth author, and Dr. Canfield should be listed as the sixth author. Their affiliations are both: California National Primate Research Center, University of California Davis, Davis, California, United States of America. The citation should be: Chen EC, Yagi S, Kelly KR, Mendoza SP, Tarara RP, et al. (2011) Cross-Species Transmission of a Novel Adenovirus Associated with a Fulminant Pneumonia Outbreak in a New World Monkey Colony. PLoS Pathog 7(7): e1002155. doi:10.1371/journal.ppat.1002155. The contributions of both authors are as follows: Performed the experiments, analyzed the data, contributed reagents/materials/analysis tools.

