# Supplementary figures and images for: Cross-Species Transmission of a Novel Adenovirus Associated with a Fulminant Pneumonia Outbreak in a New World Monkey Colony
Source: PLoS Pathog. 2011 Jul 14;7(7):e1002155. doi: 10.1371/journal.ppat.1002155 (PMC3136464; doi:10.1371/journal.ppat.1002155)

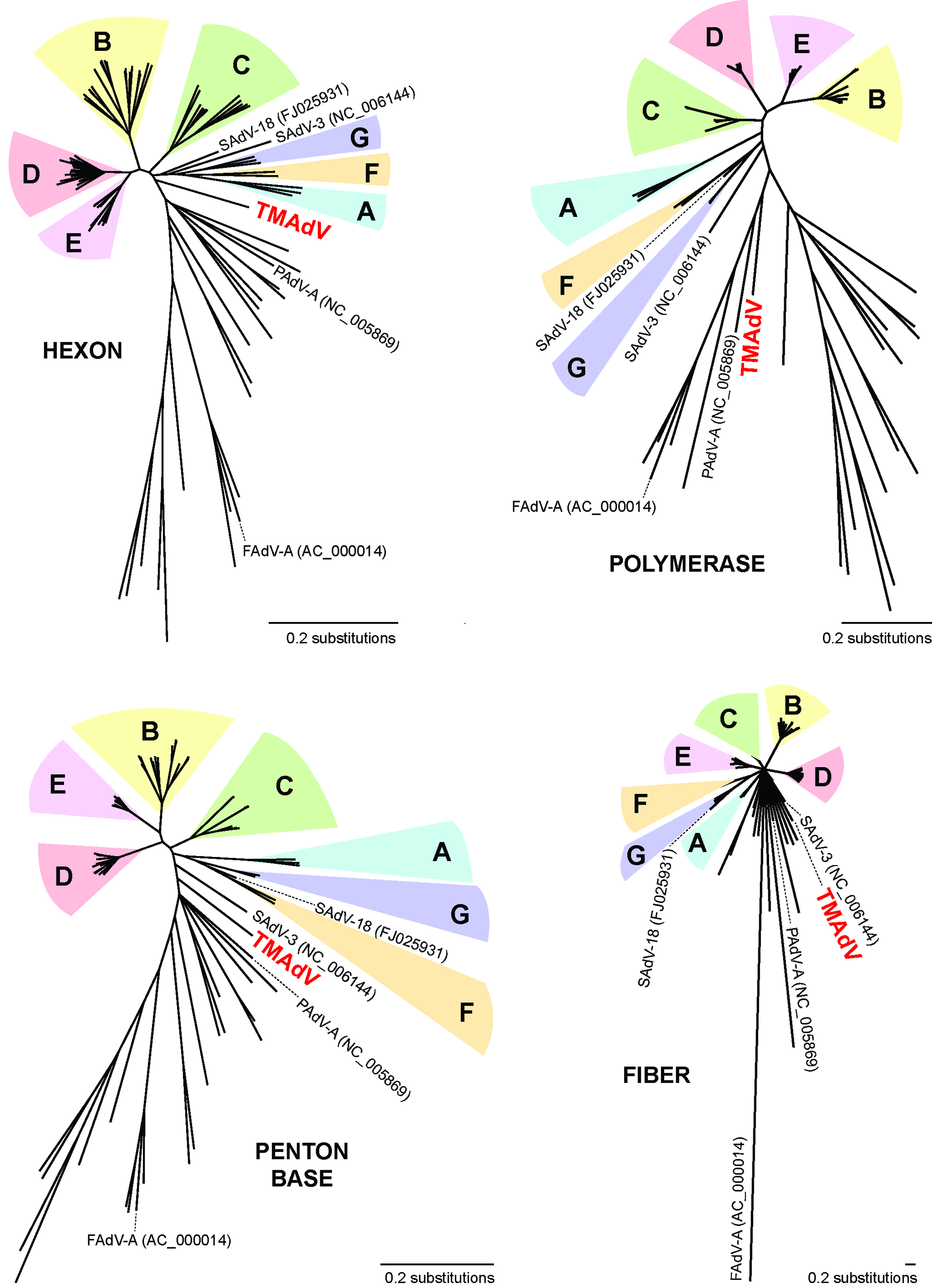

Supplement: Figure S1 — Phylogenetic analysis of the hexon, polymerase, penton base, and fiber genes of TMAdV. A multiple sequence alignment of selected genes from all 95 unique, fully-sequenced adenovirus genomes in GenBank and TMAdV is performed and the results displayed as a radial phylogenetic tree. The branch corresponding to TMAdV is highlighted in boldface red. Abbreviations: HAdV, human adenovirus, SAdV, simian adenovirus; PAdV, porcine adenovirus; FAdV, fowl adenovirus. (TIF) [file ppat.1002155.s001.tif]

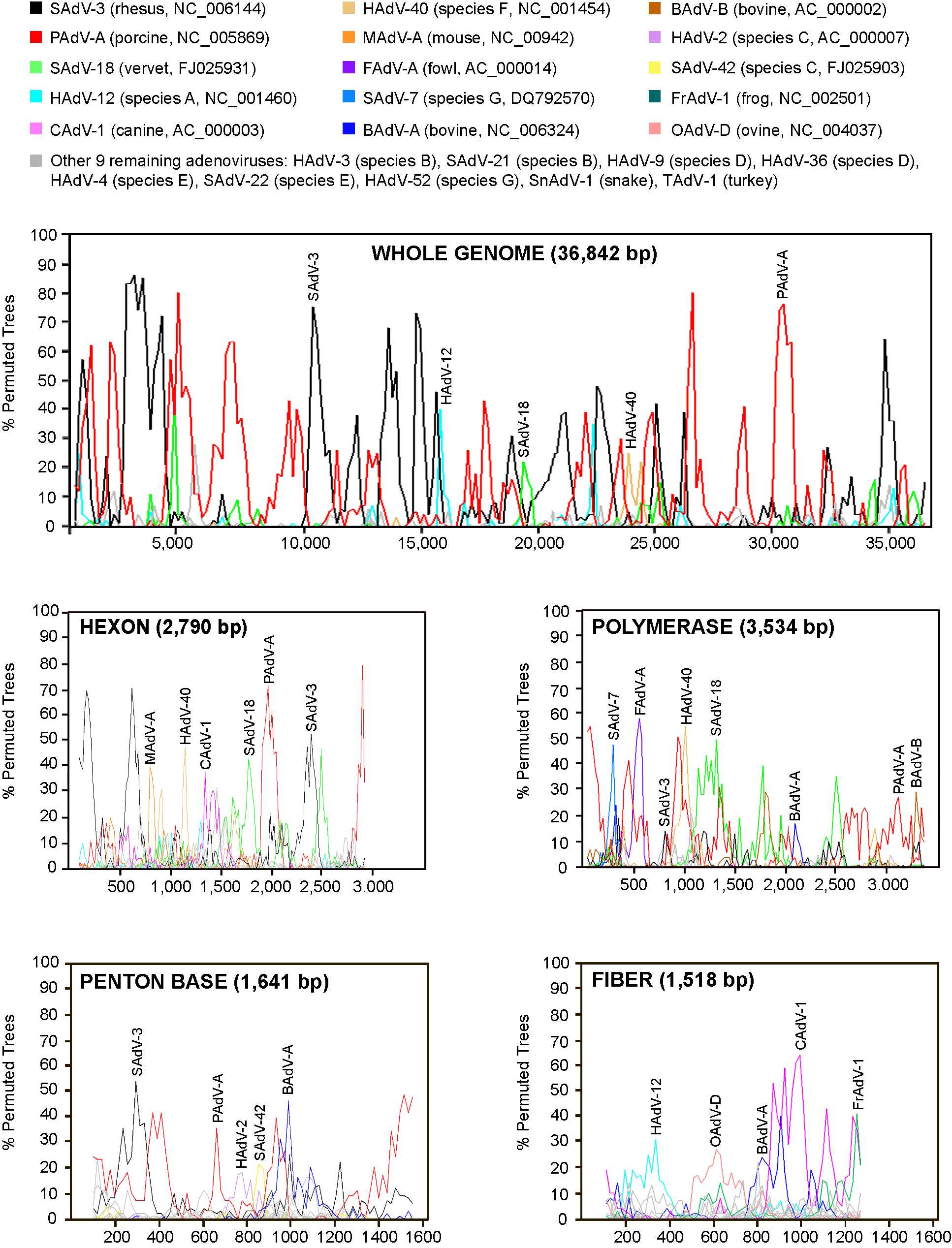

Supplement: Figure S2 — Bootscanning recombination analysis of TMAdV. Bootscanning analysis was initially performed with all 95 unique, fully-sequenced adenovirus genomes in GenBank (data not shown). After removal of similar viral genomes, bootscan plots of the whole genome and individual genes from a subset representing human/simian adenoviruses in species A–G and all non-primate vertebrate adenoviruses were generated. The window size is 400 bp with a step size of 40 bp for the whole genome, and 200 bp with a step size of 20 bp for the individual genes. The x-axis refers to the nucleotide position. For definition of abbreviations, please refer to Fig. 3. (TIF) [file ppat.1002155.s002.tif]
